# Consistent Surgeon Evaluations of Three-Dimensional Rendering of PET/CT Scans of the Abdomen of a Patient with a Ductal Pancreatic Mass

**DOI:** 10.1371/journal.pone.0075237

**Published:** 2013-09-24

**Authors:** Matthew E. Wampole, John C. Kairys, Edith P. Mitchell, Martha L. Ankeny, Mathew L. Thakur, Eric Wickstrom

**Affiliations:** 1 Department of Biochemistry and Molecular Biology, Thomas Jefferson University, Philadelphia, Pennsylvania, United States of America; 2 Department of Surgery, Thomas Jefferson University, Philadelphia, Pennsylvania, United States of America; 3 Department of Medical Oncology, Thomas Jefferson University, Philadelphia, Pennsylvania, United States of America; 4 Academic and Instructional Support and Resources, Thomas Jefferson University, Philadelphia, Pennsylvania, United States of America; 5 Department of Radiology, Thomas Jefferson University, Philadelphia, Pennsylvania, United States of America; 6 Kimmel Cancer Center, Thomas Jefferson University, Philadelphia, Pennsylvania, United States of America; The University of Chicago, United States of America

## Abstract

Two-dimensional (2D) positron emission tomography (PET) and computed tomography (CT) are used for diagnosis and evaluation of cancer patients, requiring surgeons to look through multiple planar images to comprehend the tumor and surrounding tissues. We hypothesized that experienced surgeons would consistently evaluate three-dimensional (3D) presentation of CT images overlaid with PET images when preparing for a procedure. We recruited six Jefferson surgeons to evaluate the accuracy, usefulness, and applicability of 3D renderings of the organs surrounding a malignant pancreas prior to surgery. PET/CT and contrast-enhanced CT abdominal scans of a patient with a ductal pancreatic mass were segmented into 3D surface renderings, followed by co-registration. Version A used only the PET/CT image, while version B used the contrast-enhanced CT scans co-registered with the PET images. The six surgeons answered 15 questions covering a) the ease of use and accuracy of models, b) how these models, with/without PET, changed their understanding of the tumor, and c) what are the best applications of the 3D visualization, on a scale of 1 to 5. The six evaluations revealed a statistically significant improvement from version A (score 3.6±0.5) to version B (score 4.4±0.4). A paired-samples t-test yielded t(14) = −8.964, p<0.001. Across the surgeon cohort, contrast-enhanced CT fused with PET provided a more lifelike presentation than standard CT, increasing the usefulness of the presentation. The experienced surgeons consistently reported positive reactions to 3D surface renderings of fused PET and contrast-enhanced CT scans of a pancreatic cancer and surrounding organs. Thus, the 3D presentation could be a useful preparative tool for surgeons prior to making the first incision. This result supports proceeding to a larger surgeon cohort, viewing prospective 3D images from multiple types of cancer.

## Introduction

Surgical evaluation of a potential pancreatectomy is a complicated task requiring three-dimensional (3D) spatial awareness of the tumor impact on surrounding normal structures, including the vasculature, pancreas, and spleen. This assessment will determine whether or not a resection should be attempted. If the lesion is operable, the orientation of the pancreas and its surrounding structures will guide planning of a detailed strategy for the surgical intervention. Typically the decision and planning steps are based upon two-dimensional (2D) images of the patient from a variety of sources, including computed tomography (CT), magnetic resonance imaging (MRI), ultrasound, and positron emission tomography (PET) [1], [2], [3], [4]. Planning for this delicate operation requires surgeons to translate the 2D image slices into a 3D mental picture of the patient’s anatomy, which can be a difficult task.

CT images are a widely used, non-invasive method to study bone and tissue structures for diagnostic and therapeutic purposes. The 2D images produced are arrayed in a stack of slices along the sagittal, axial, and coronal planes of the subject. The pixel intensities are dependent on the how various structures within the patient attenuate the X-Ray beam: high density regions will appear bright while low density regions will appear darker. Images taken by CT can be enhanced with the use of an intravenous contrast agent to improve the image intensities of internal organs, particularly the blood vessels. Contrast enhancement is useful for pre-operative planning of pancreatic cancer patients since a number of vessels can be impacted.

**Figure 1:**
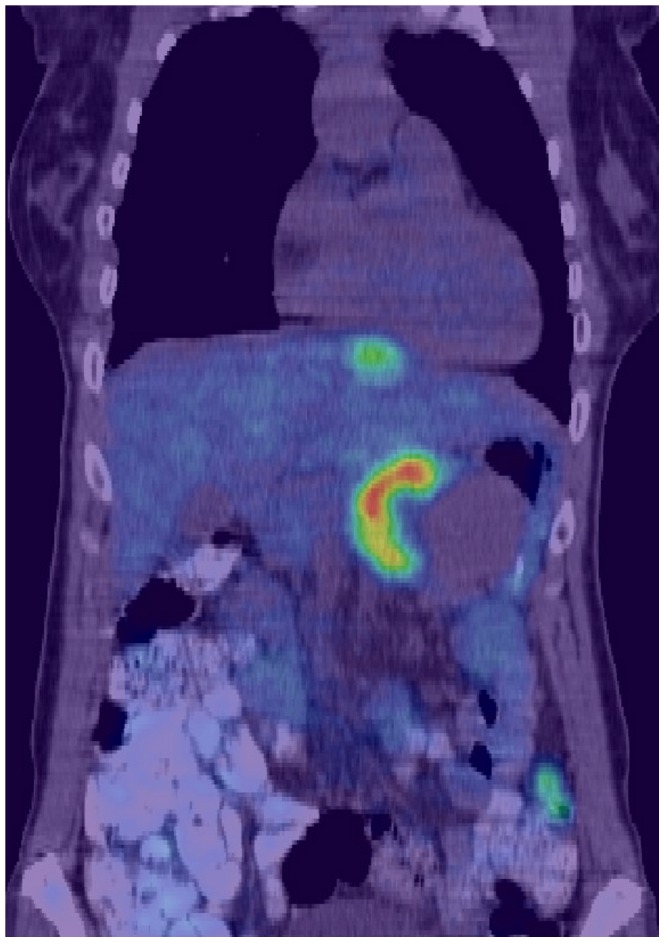
2D CT/PET fusion image slice of an anonymized patient with a ductal pancreatic mass. Regions with the highest 2′-[^18^F]fluorodeoxyglucose emission are colored red here while the lowest emissions are colored blue. A surgeon currently looks back and forth through a stack of such images to gain an understanding of anatomy surrounding the lesion. For this image in the coronal plane, the displayed PET window was narrowed to accentuate the location of high uptake in the pancreas as well as another hotspot in the liver.

PET (Figure 1) makes imaging of tissues of interest possible by injecting a biologically active positron-emitting radiolobel into the patient and detecting where it accumulates. The specificity of the radiolabel is important for accurately marking a target tissue for imaging. In particular, PET imaging with 2′-[^18^F]fluorodeoxyglucose (^18^FDG PET) has become a useful tool in the evaluation of tumor lesions, particularly for pancreatic cancer, and the discovery of distant metastases [5], [6], [7], [8]. ^18^FDG is a glucose analog that is taken up avidly by rapidly growing malignant masses, but not all hot spots are cancerous. Inflamed or infected tissues also accumulate ^18^FDG. The kidneys filter out excess glucose from the blood, which results in strong ^18^FDG images of the kidneys. Likewise the bladder also shows a strong PET image due to accumulation of ^18^FDG waiting to be eliminated.

While CT and PET are useful imaging modalities on their own, combining them can yield new insights into the nature of the lesion and surrounding structures. When combining multiple modalities, the images must be registered with each other to form a single frame of reference [9].

Manual rendering of internal organs as 3D surfaces can be time-intensive. The essential step in visualizing the patient’s anatomy in 3D is segmentation, which assigns pixels of the image to a particular organ. Automating this method has been attempted for specific organs such as the liver [10], [11], breast [12], bladder, lungs [13], or blood vessels [14]. These methods are complicated by boundaries between organs that are of similar intensities. The pancreas, in particular, requires some manual effort to accurately define the boundaries [15], [16], [17], [18].

3D visualization is the first step into new methods of anatomical analysis. Simulations of biological structures are of growing interest for pre-surgical planning, computer-aided surgery, and teaching aids [19], [20]. These simulations require accurate representation of patient physiology and incorporation of other image sets to improve diagnostic accuracy [21]. Combining multiple modalities into into individual patient 3D presentations is intended to improve the surgeon’s ability to prepare for a procedure [22]. We hypothesized that experienced surgeons could consistently evaluate the usefulness of a 3D visualization of patient CT images co-registered with PET images as a pre-operative assessment tool.

## Materials and Methods

### Ethics Statement

Anonymous use of patient CT and PET data, and questionnaires for surgeons evaluating usefulness, were approved by the TJU IRB (09E.407) and the USAMRMC HRPO (A-15712.2). Files with patient data were anonymized before data manipulation began. The main criteria for inclusion into this study were a large pancreatic mass, an abdominal PET/CT image, and an abdominal IV contrast diagnostic CT.

### Surgeons

Two surgical oncologists and four general surgeons from Thomas Jefferson University Hospital volunteered to evaluate two versions of the patient 3D abdominal visualization. All routinely perform pancreatic resections, with one to 26 years of experience post-residency or post-fellowship. The surgeons were shown how to manipulate the 3D model, especially how to strip off tissues and organs overlying the pancreas.

### Evaluations

The evaluation consisted of fifteen questions using a scale from 1 to 5∶1 =  strongly disagree, 2 =  disagree, 3 =  neither agree/disagree, 4 =  agree, and 5 =  strongly agree. The questions were categorized into three sections: (i) ease of use and accuracy of the models, (ii) how these models, with/without PET, changed their understanding of the tumor, and (iii) useful applications of the 3D visualization. Blank space was also provided for each surgeon to leave comments on the visual appearance and usefulness of the presentation, plus any overall comments.

The evaluation and questionnaire were done in the presence of the interviewer to help answer any questions about the visuals, manipulating Amira 5, or the questionnaire. Amira 5 (Visage Imaging, San Diego CA), is a 3D visualization, analysis and modeling program that takes a modular and object-oriented approach to data visualization and analysis This mode facilitated the surgeons’ usage of Amira 5, independent of any prior knowledge of the program, and yielded a more reliable evaluation of the system.

### Statistical Analysis

Significance of the questionnaire responses was analyzed with SigmaPlot 11. The surgeon scores for each question were averaged, and the standard deviation was calculated. A paired-samples t-test was carried out to compare the average of the scores given to each question in version A and version B.

### CT and PET Scans

One female patient, age 51, met the criteria specified. Three sets of data for the patient were collected for visualization: one non-contrast CT at 1 mm (512×512) resolution and 2 mm slice thickness on a Siemens Biograph 6 (Siemens Medical Solutions USA, Inc, Malvern, PA) PET/CT scanner, one PET image at 5 mm (128×128) resolution and 2.5 mm slice thickness co-registered with the non-contrast CT, and one intravenous contrast CT at 0.5 mm (512×512) resolution and 1.5 mm slice thickness using a Philips Brilliance 16P CT scanner.

### 3D Visualizations

Transfering the image information in the 2D CT and PET image slices into 3D surfaces of the patient’s abdomen were carried out with Amira 5. Two versions of the visualization were developed for subsequent evaluation by surgeons.

The first version (A) was based solely upon the PET/CT images and had no surface refinement. The segmentation of this dataset did not include a complete vascular network, but required no registration of the PET image, since they were already pre-aligned. After evaluation of version A, the second version (B) was developed using the contrast CT images, which included a more complete vascular network than version A. This version required that the PET image be aligned with the contrast CT image.

### Image Segmentation

Segmentation of the stack of 2D CT image slices was accomplished semi-automatically using a mixture of the Amira 5 ‘magic wand’ tool, the ‘blow tool’, and manual segmentation. Segmentation of each image slice in the stack yields a matrix of points, or label field, which defines which pixels are associated with each other.

The ‘magic wand’ tool uses a region growing function. When the user selects a voxel, an area containing that voxel and any number of other voxels whose intensities lie within the user’s defined ranges are selected. Lines can be drawn to limit the extent of the growth. Similarly, the ‘blow tool’ was another region growing method that increases as the cursor moves away from the initially clicked point. The region grows in area of similar intensities, stopping where the values change abruptly, i.e., edges.

The segmented images were then refined using the ‘smooth label’ and ‘remove islands’ options. The ‘smooth label’ tool uses a modified Gaussian filter to smooth the regional boundaries, removing any cusps that appear in the surface. The ‘remove islands’ tool finds isolated regions not connected to the larger region and removes them from the segmented label. The ‘remove islands’ tool can also find holes within the region and add them to the label.

Segmenting of the PET images was done with the ‘magic wand’ tool. The high contrast between the ^18^FDG uptake region of the tumor vs. the surrounding normal tissue made it possible to choose a threshold that would pick only regions of high intensity. The first author performed these segmentations with no previous experience in medical segmentation and the second author, a surgeon with over 18 years of experience reviewed these structures for accuracy. It took approximately two days to segment a single CT data set from beginning to end.

### Surface Rendering

Each stack of fully segmented patient abdominal image slices was rendered as a 3D surface with an Amira 5 SurfaceGen module attached to each 2D label field. The SurfaceGen module computes a triangular approximation of the surface from the 2D label fields and the interfaces between differing regions. These base 3D visuals have a large number of triangles and can appear rather rough. One example would be the liver, consisting of 129,325 points and 258,622 triangles. The entire rendering of the patient had 1,216,386 triangles over 12 models: skeleton, liver, intestines, duodenum, right kidney, left kidney, stomach, spleen, pancreas, adrenal glands, veins, and arteries. As an initial step in smoothing the visuals, the ‘smoothing’ option of the SurfaceGen module was set to unconstrained smoothing and the SmoothKernelSize variable was set to 3.

### Surface Quality and Refinement

The Amira 5 Simplification editor was applied to the abdominal base 3D visuals to improve the quality and reduce the number of triangles forming the visuals. The Simplification editor uses an edge collapsing algorithm to reduce edges of the surface to points, while preserving the original shape of the surface by minimizing the error criterion. The 3D surfaces were then re-meshed to improve their appearance using the RemeshSurface module. For remeshing, an isotropic vertex placement and a 50% reduction in the number of triangles was used to achieve a higher triangle quality and modest reduction in the number of triangles. At this point the liver was simplified to 9002 points and 18000 triangles before being remeshed to 4501 points and 8998 triangles. In version B, the entire patient rendering contained 225,221 triangles with the same number of organs being represented.

### Registration

Registration of multiple images is necessary for studies of a subject over time, when comparing different modalities, matching an image surface with its model, and aligning a template with the patient’s image. Aligning multiple images correctly is a difficult task due to organ motion during respiration, patient re-positioning, organ changes over time due to disease, deformation of target organs, and many more. Automated registration exists for certain applications, such as the lung or arteries, but is not amenable for use with the pancreas, due to similar intensities in the voxels between the pancreas and neighboring organs.

A hybrid of automated and interactive registration was used with the PET/CT, CT-only, and contrast CT data sets. The focus of the registration was to align the PET images of the pancreas so that they could be overlaid with the contrast CT model. The CT dataset without contrast, which was initially aligned with the PET images, was aligned to the contrast CT before applying the transformation to the PET images. Both the CT data and the 3D renderings were used for the registration.

Initially, automated registration with the AffineRegistration module aligned the images roughly. The AffineRegistration module uses the mean squared differences between gray values of the model and reference as the metric for alignment and scaling, prior to further refinement. Interactive registration was then carried out to refine the alignment and scale of the images. Amira 5 provides three parameters in the x, y, and z directions with which to adjust the image transformation; translation, rotation axis, and scale factor.

The final stage of registration of the 3D surfaces of the pancreas utilized the AlignSurface module. This module has three strategies for aligning the surface, using the surface points, the center of mass, or the principal axes of the inertia tensor. Three types of transformation can be specified: rigid alignment, rigid alignment with uniform scaling, or a flexible affine transformation. We used the surface points with a rigid alignment to align the pancreas of the non-contrast CT with that of the contrast CT.

Once the two renderings were aligned with each other, the transformation could be applied to the PET images. Since the PET images were initially aligned with those of the non-contrast CT, the transformations applied to the CT images were repeated with the PET images.

## Results

### 3D Visualizations

**Figure 2:**
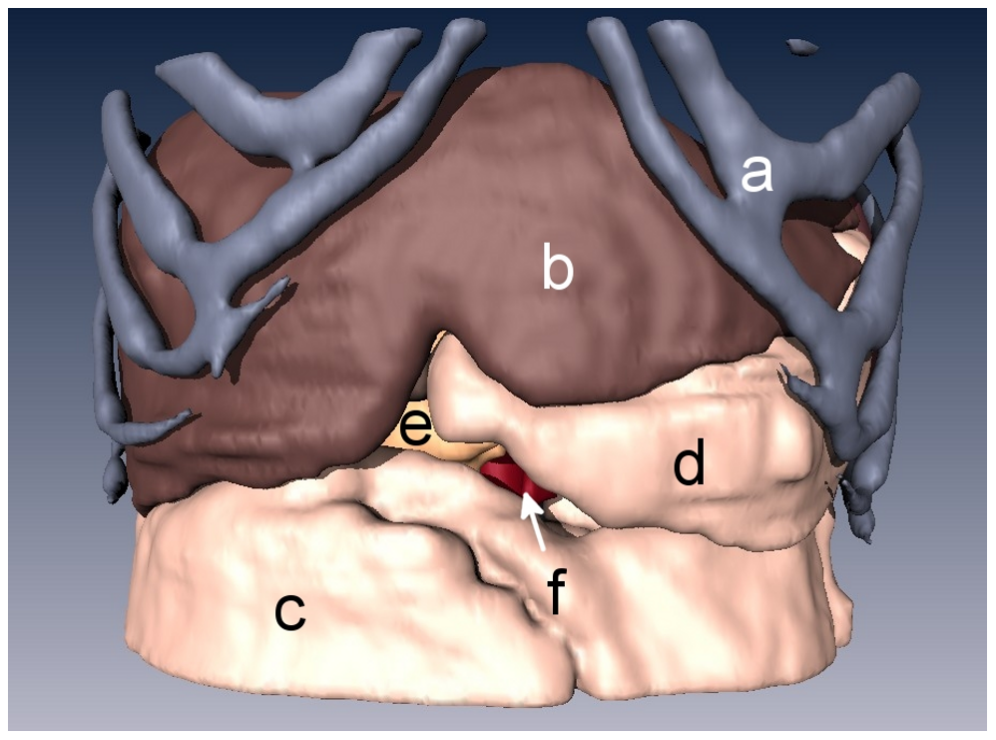
3D rendering of an anonymized patient’s abdomen with a ductal pancreatic mass, version A. Organs displayed in this rendering include **a)** rib cage, **b)** liver, **c)** intestines, **d)** stomach, **e)** pancreas, and **f)** aorta.

**Figure 3:**
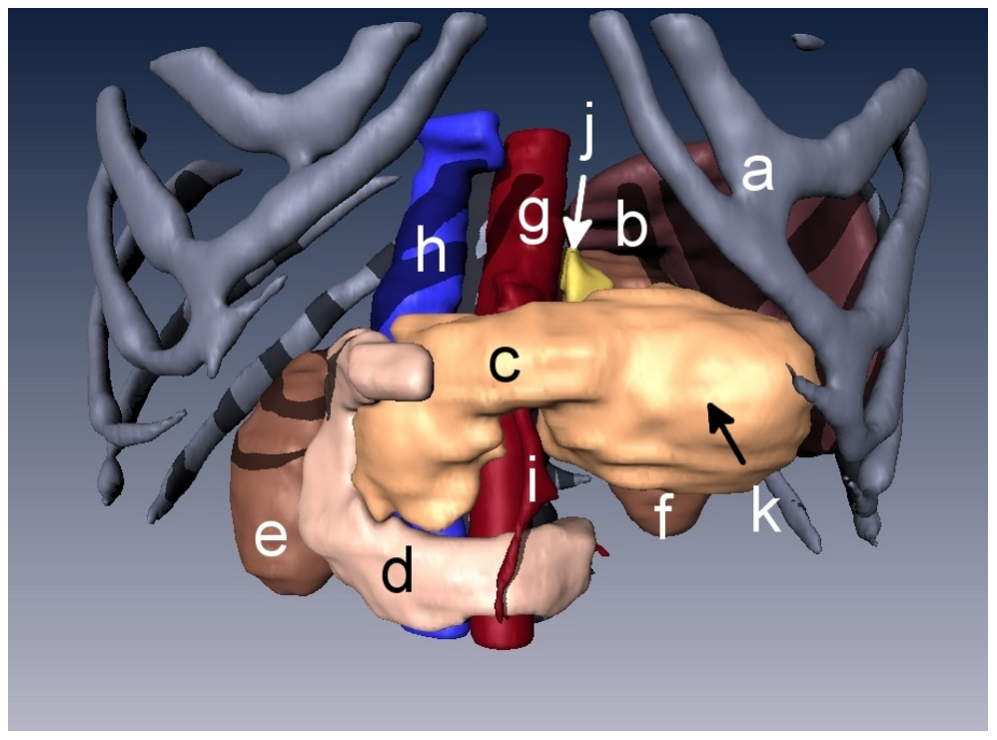
3D rendering of the abdomen with organs stripped away to display the pancreatic tumor. The liver, stomach, and intestines were not visualized for a clearer view of the pancreas and its surroundings. Organs displayed in this rendering include **a)** rib cage, **b)** spleen, **c)** pancreas, **d)** duodenum, **e)** right kidney, **f)** left kidney, **g)** aorta, **h)** vena cava, **i)** superior mesenteric artery, **j)** adrenal gland, and **k)** pancreatic tumor.

**Figure 4:**
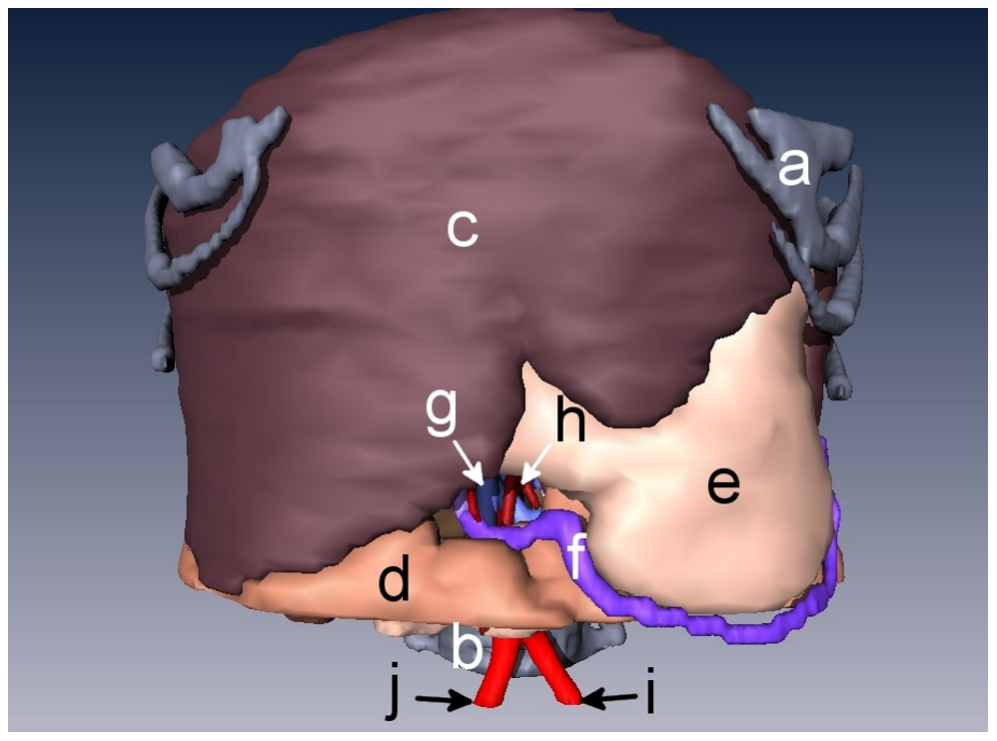
3D rendering of an anonymized patient’s abdomen with a ductal pancreatic mass, version B. Organs displayed in this rendering include **a)** rib cage, **b)** spine, **c)** liver, **d)** intestines, **e)** stomach, **f)** right gastroepiploic vein, and **g)** superior mesenteric vein, **h)** superior mesenteric artery, **i)** left common iliac artery, and **j)** right common iliac artery.

**Figure 5:**
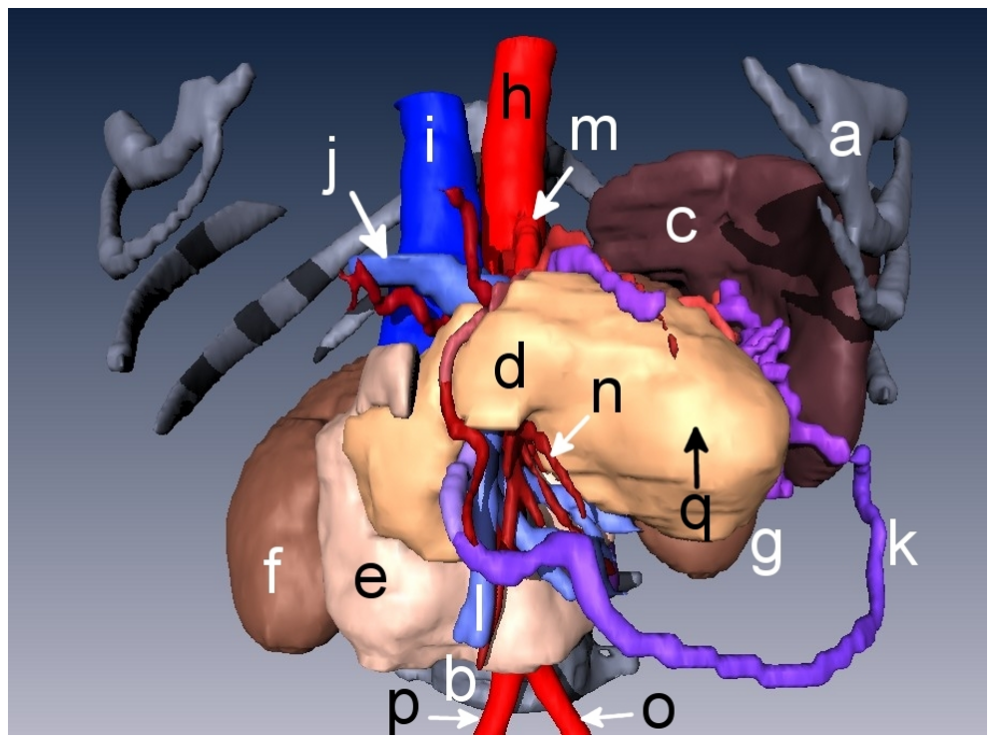
3D rendering of the abdomen with organs stripped away to display the pancreatic tumor. The liver, stomach, and intestines were not visualized for a clearer view of the pancreas. Organs displayed in this rendering include **a)** rib cage, **b)** spine, **c)** spleen, **d)** pancreas, **e)** duodenum, **f)** right kidney, **g)**, left kidney **h)** aorta, **i)** vena cava, **j)** portal vein, **k)** right gastroepiploic vein, **l)** superior mesenteric vein, **m)** celiac artery, **n)** superior mesenteric and intestinal arteries **o)** left common iliac artery, **p)** right common iliac artery, and **q)** pancreatic tumor.

**Figure 6:**
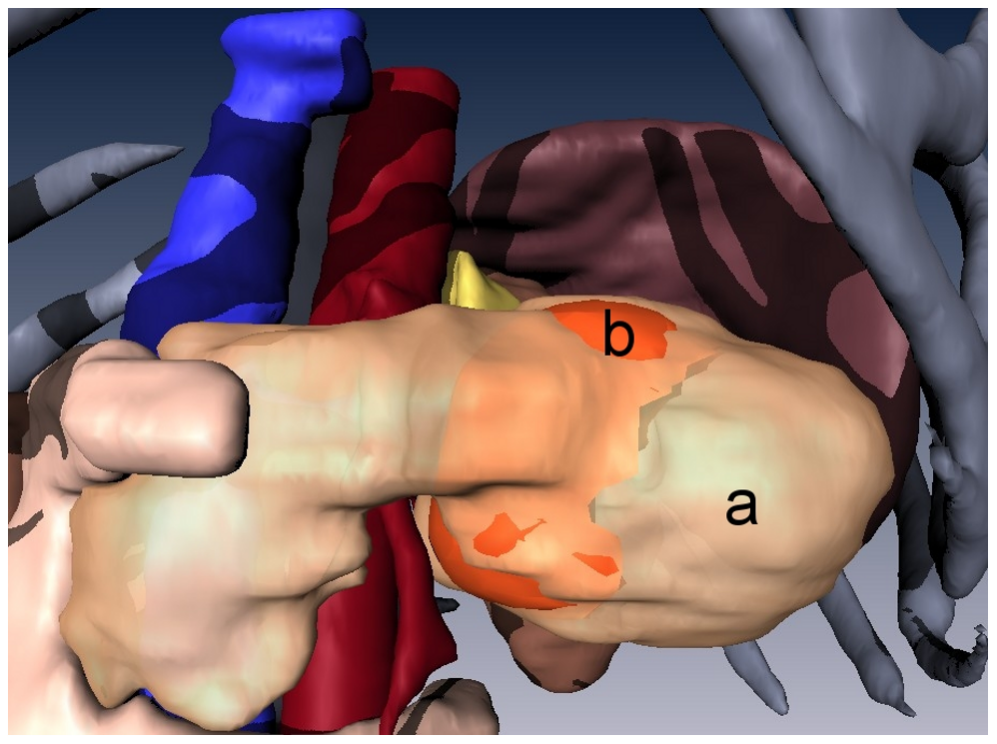
3D rendering of the ductal pancreatic mass, version A, fused with PET data. A transparent overlay of (**a**) the pancreas over (**b**) the surface rendering of the high ^18^FDG uptake region of the pancreatic tumor (orange).

Snapshots from version A of the 3D abdominal visualizations are shown in Figures 2 and 3, while snapshots from version B appear in Figures 4 and 5. An example of the ^18^FDG -PET overlay is given in Figure 6. Because of the lower resolution in the PET images (5 mm), parts of the surface representation of the tumor formed from the PET data do not exactly replicate the contours of the pancreas.

### Overall Evaluations

The participating surgeons evaluated the 3D renderings by questionnaire to determine which aspects and features of the abdominal visualizations they found useful. For each question, 5 was the highest rating, and 1 was the lowest rating. There was an overall improvement in the ratings of the visualizations going from version A to version B.

**Figure 7:**
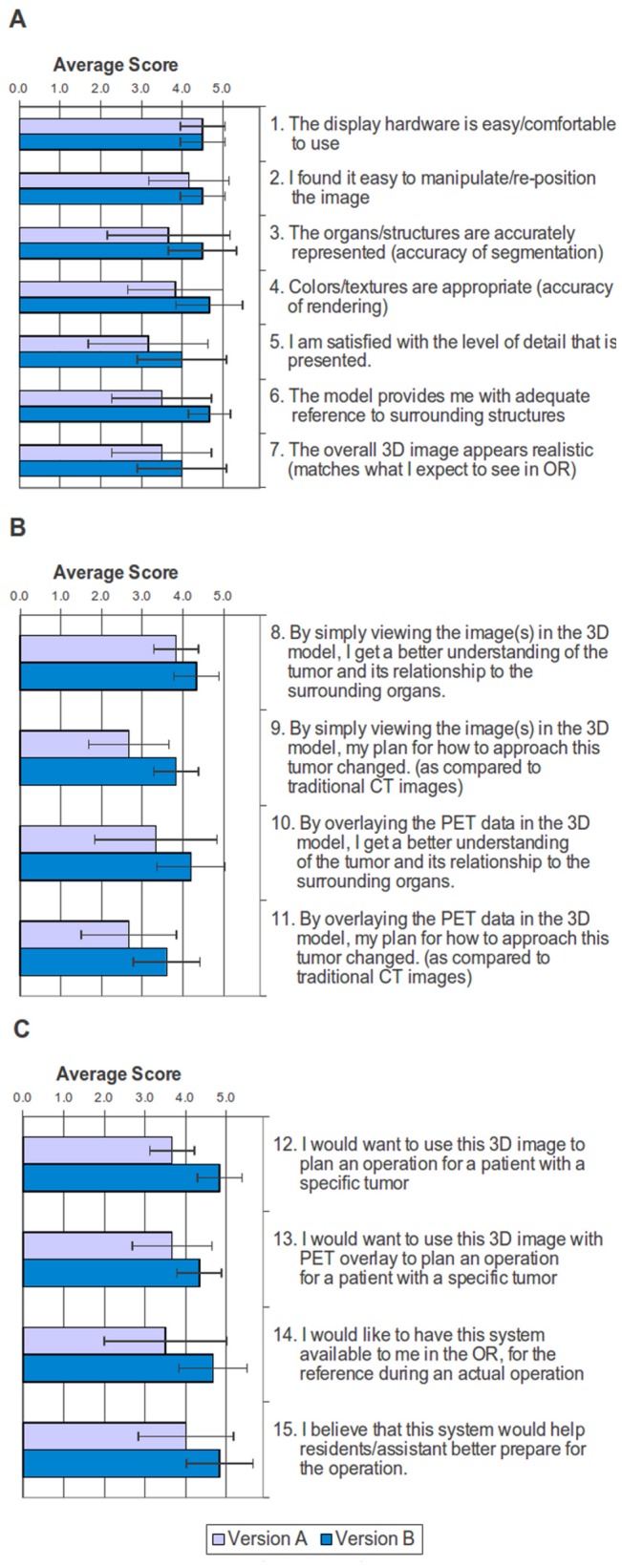
Average surgeon evaluation scores for version A and version B of the 3D visualization. Average scores, with error bars, are shown for questions on **a)** the ease of use and accuracy of models, **b)** how these models, with/without PET, changed their understanding of the tumor, and **c)** what are the best applications of the 3D visualization.

Figure 7 presents the average scores from the surgeon evaluations of versions A and B for each of the 15 questions. Averaging surgeon responses to these questions, version A scored 3.6±0.5, while version B received a score of 4.4±0.4. The overall averages of the questionnaire illustrate greater appreciation of version B, while the standard deviations reveal greater consensus in evaluating version B. A paired-samples t-test detected a statistically significant difference between the scores for version A and version B: t(14) = −8.964, p<0.001. The questionnaire responses show that the improvements in version B had a positive impact on the scoring of the visualizations.

### Ease of use and Accuracy of Models

In both versions, the surgeons found the hardware and image manipulation/re-positioning easy to use. Hardware was assigned an average score of 4.5±0.6 for both versions. Image manipulation scores increased from 4.2±1.0 for version A to 4.5±0.6 for version B.

The accuracy of the segmentations, their rendering, and the surgeons’ satisfaction with the level of detail all improved going from version A to version B. For version A, 4 out of 6 surgeons agreed with the accuracy of the segmented images, with one neither agreeing nor disagreeing, and one strongly disagreeing, for an average score of 3.7±1.5. The colors and textures used were quite similar with 4 of 6 agreeing, one neither agreeing nor disagreeing, and one disagreeing, for an average score of 3.8±1.2. The average score for the level of detail was 3.2±1.5 with only half the surgeons being satisfied with it, one neither agreeing nor disagreeing, one disagreeing, and the last strongly disagreeing.

Version B, on the other hand, received higher scores of 4.5±0.8, 4.7±0.8, and 4.0±1.1 for the segmentation, rendering, and level of detail. Only one surgeon couldn’t agree or disagree with the segmentations and rendering, but disagreed with the level of detail.

Overall the surgeons assigned version A a score of 3.5±1.2 for the model providing adequate reference to surrounding structures, and a score of 3.5±1.2 for how well it matches expectations in the operating room while version B faired much better with scores of 4.7±0.5 and 4.0±1.1, respectively.

### How these Models, with/without PET, Changed their Understanding of the Tumor

While viewing only the 3D CT renderings of the patient’s abdomen, the surgeons were asked to rate their understanding of the tumor with its surrounding organs and if this view would change how they might plan to approach this tumor. Version A scored an average of 3.8±1.2 on understanding of the tumor and its surroundings, but a 2.7±1.0 on changing how they might plan to approach it. For version B, however, average scores of 4.3±0.8 and 3.8±0.8 were given for understanding of the tumor with its surrounding organs, and if this view would change how they might plan to approach this tumor, respectively.

Viewing the 3D CT renderings with the PET overlay was rated similarly to those without the overlay for understanding of the tumor with its surrounding organs, and if this view would change how they might plan to approach this tumor. Version A received scores of 3.3±1.0 and 2.7±1.2 for understanding and approach, while version B was scored at 4.2±0.8 and 3.6±0.9.

### Best Applications of the 3D Visualization

Four questions were asked on the usefulness of the visualizations. The first question asked if the surgeon would want to use this 3D image to plan an operation for a patient with this specific tumor. The surgeons scored version A at 3.7±1.8, and version B at 4.8±0.4. In the second question, the surgeons were asked if they would want to use these images with the PET overlay to plan an operation for a patient with this specific tumor. They scored version A at 3.7±1.8, and version B at 4.3±1.2. Next, the surgeons were asked if they would like a system such as this available in the operating room for reference during an actual operation. For version A the response was neutral with a score of 3.5±1.6, but version B received strong agreement with an average of 4.7±0.5. The final question asked if this system would help residents/assistants better prepare for the operation. In both versions the surgeons agreed, giving version A a score of 4.0±1.6 and version B a score of 4.8±0.4.

### Additional Comments

Each surgeon was given space on each questionnaire to leave comments on how the visual models could be improved and what would make the models more useful. In version A every surgeon who commented asked for a more detailed inclusion of the vascular system. Suggestions were left for more textures and detail in the models. With version B, an appreciation for the inclusion of the vascular system was seen in the comments. Three asked for improvements in the fine details of the tumor and blood vessels or better resolution in the images presented. In this version the blood vessels were given slightly different hues to help differentiate the branches. One surgeon proposed making all the vessels one color, while another wanted to be able to remove specific vessels as desired. One other comment mentioned that tumor invasion of adjacent structures was difficult to determine.

## Discussion

An ideal preoperative assessment of pancreatic cancer would provide accurate definition of the relationship of the malignant tissue with associated normal structures. Combining anatomical imaging with molecular imaging would be useful for presurgical staging and planning, altering disease management to minimize complications and reveal occult lesions [23].

For the 3D PET/CT imaging method presented above, surgeon evaluations of the two versions were positive overall, but the improvements in version B yielded much better scores. Using the contrast enhanced CT slices to develop version B provided a more detailed vascular system, contributing the most to score improvement.

**Figure 8:**
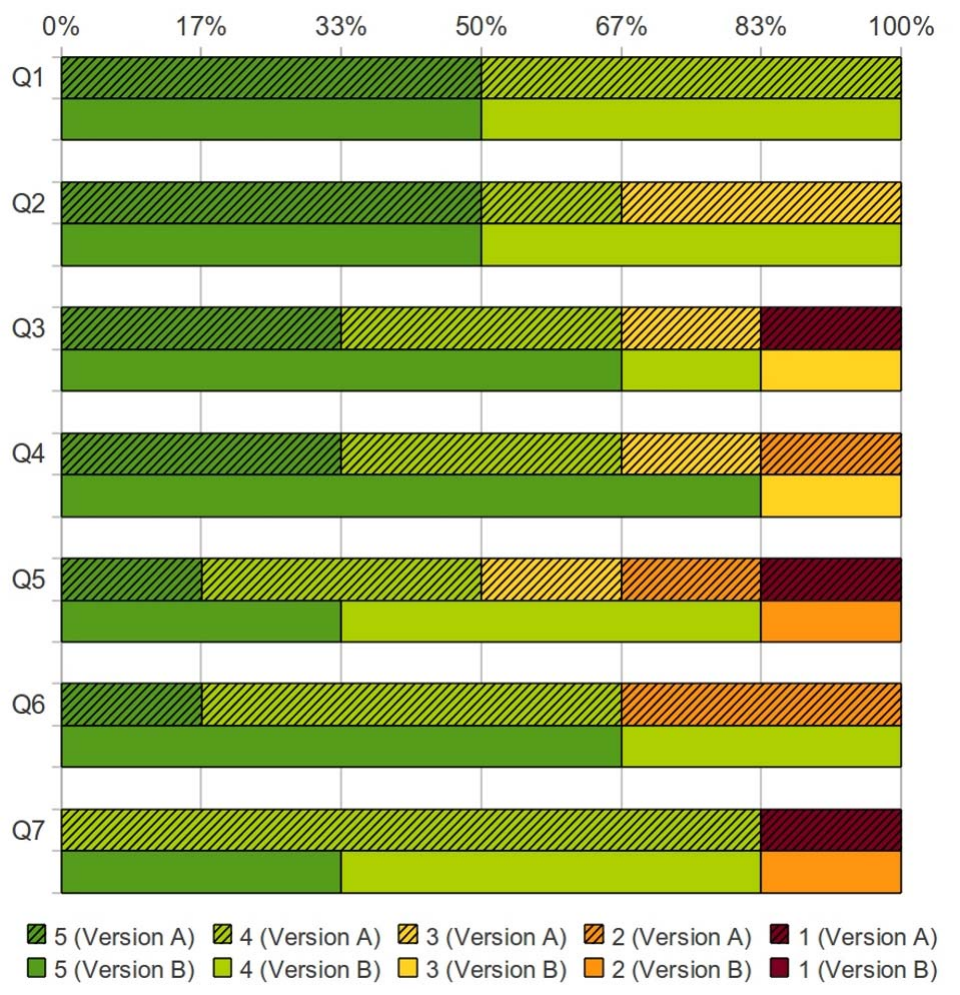
Score distribution on the ease of use and accuracy of models. Percentages of surgeons who assigned a particular score for both Version A (solid colors with hatch marks) and Version B (solid colors) [Q1: The display hardware is easy/comfortable to use, Q2: I found it easy to manipulate/re-position the image, Q3: The organs/structures are accurately represented (accuracy of segmentation), Q4: Colors/textures are appropriate (accuracy of rendering), Q5: I am satisfied with the level of detail that is presented, Q6: The model provides me with adequate reference to surrounding structures, Q7: The overall 3D image appears realistic (matches what I expect to see in the OR)].

**Figure 9:**
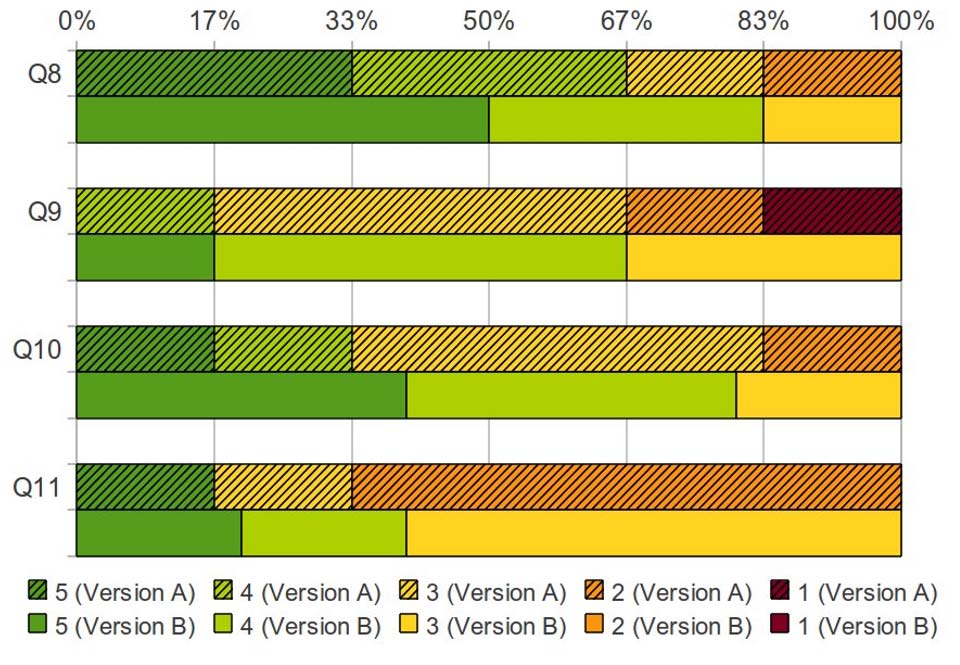
Score distribution on how these models, with/without PET, changed their understanding of the tumor. Percentages of surgeons who assigned a particular score for both Version A (solid colors with hatch marks) and Version B (solid colors) [Q8: By simply viewing the image(s) in the 3D model, I get a better understanding of the tumor and its relationship to the surrounding organs, Q9: By simply viewing the image(s) in the 3D model, my plan for how to approach this tumor changed (as compared to traditional CT images), Q10: By overlaying the PET data in the 3D model, I get a better understanding of the tumor and its relationship to the surrounding organs, Q11: By overlaying the PET data in the 3D model, my plan for how to approach this tumor changed (as compared to traditional CT images)].

**Figure 10:**
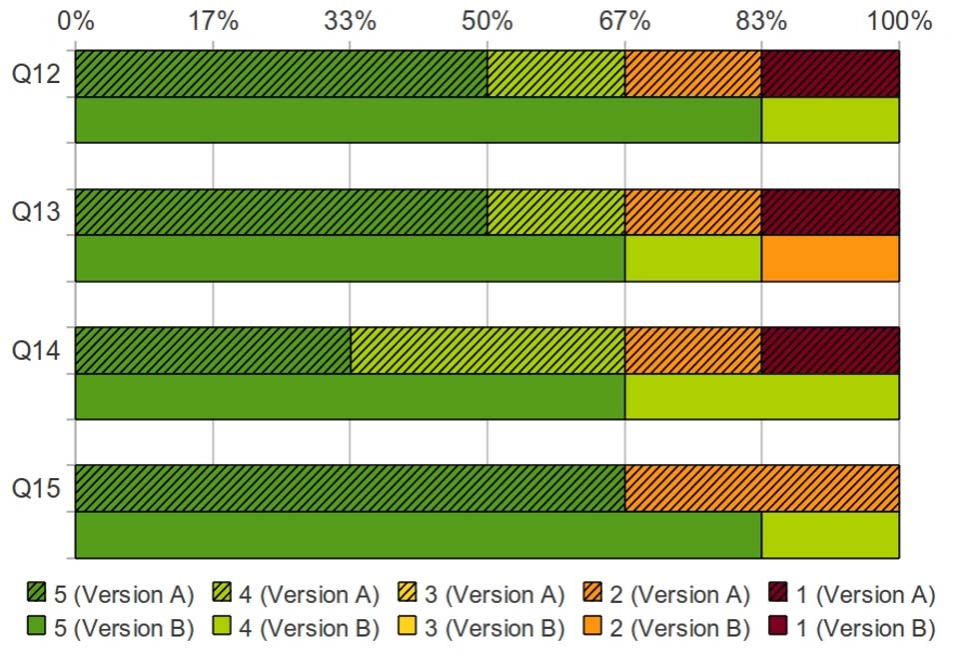
Score distribution on what are the best applications of the 3D visualization. Percentages of surgeons who assigned a particular score for both Version A (solid colors with hatch marks) and Version B (solid colors) [Q12: I would want to use this 3D image to plan an operation for a patient with a specific tumor, Q13: I would want to use this 3D image with PET overlay to plan an operation for a patient with a specific tumor, Q14: I would want to have this system available to me in the OR, for the reference during an actual operation, Q15: I believe that this system would help residents/assistant better prepare for the operation].

Tumor invasion into neighboring structures is of great importance for determining whether resection is appropriate. Color-coded distributions of the scores assigned by the surgeons for version A vs. version B are shown in Figures 8, 9, and 10. Version A received twenty-three disagreeing scores, while version B received only three disagreeing scores.

The positive responses from the surgeon questionnaires invites further study of the usefulness of the 3D visualization method over a larger set of surgeons, patients, and lesions, including such variables as surgeon experience, tumor extension, or tumor size. A followup investigation should also identify how the improvements affected each question individually, requiring a larger cohort of evaluators.

The time required to manually segment the complicated portions, the duodenum and pancreas, of the patient’s anatomy identified a shortcoming of the current procedure. Taking two days to segment a single patient would be an undue burden in a clinical situation. Automated methods for segmentation of the pancreas are being developed [16], [17], [18].

Some of the organ surface renderings, for example the kidney in Figure 4, exhibited an unnatural stair-step appearance on some of the edges. This was caused by large changes in position between image slices that the surface rendering algorithm failed to smooth into a single curve. In general, higher resolution scans would yield a smoother image, at a cost of higher radiation doses. A more aggressive algorithm for smoothing could be used, but at a cost to the accuracy of fine structures. Additionally, each piece of anatomy may need individual care when smoothing. For example, thin objects, such as the blood vessels, will appear blockish in regions of sharp curves if a strong smoothing algorithm is applied.

The resolution of the images should also be considered when overlaying PET images with CT. In this study, the CT images had a resolution of 1 mm while the PET images had a lower resolution of 5 mm. The low resolution of PET images can introduce inaccuracy into the visualization from two partial volume effects [24]. The first phenomenon causes the PET source to bleed into neighboring regions, appearing larger than it actually is but also less intense. A second phenomenon is due to the intensity assigned to a voxel is the mean of tissues within it. Underestimating or overestimating the boundaries of the PET image could lead to the impression that the tumor is starting to invade neighboring tissues (Figure 6). Obtaining high resolution PET images or further research into ways to minimize these effects would greatly benefit in the diagnosis of tumor staging.

How the years of experience might affect the surgeons’ perceived usefulness of the visualization is one topic of interest. Long experience in using a series of standard 2D diagnostic images could result in a preference for that standard mode of pre-operative planning. On the other hand, less experienced surgeons will have less comfort or speed in analyzing the 2D images than their more experienced colleagues. Overall this might lead the surgeons with more experience to be neutral about their experience with the 3D visualizations then those with less experience. This knowledge would be a useful metric to see if 3D visualization can present as much or more information to an experienced surgeon as typical 2D images. To explore this possibility a larger cohort of evaluating surgeons with a variety of experience would be required.

The usage of 3D visualizations as opposed to the standard 2D slice held a number of advantages. Seeing the blood vessels as they wrap around the tumor is a powerfully useful tool for pre-operative planning. With 2D slices the, a surgeon must scroll through each slice and mentally reconstruct how the vessels will wrap around the organ and interact with the tumor. This is further complicated by the addition of PET data for denoting areas of increased FDG uptake. Displaying this data in 3D allowed the surgeons to view the patient data as it could appear during surgery without mental reconstruction. This could allow them to spend more time planning where obstacles are, how to handle these obstacles and where to make margins.

The choice of modality is important for accurately diagnosis of pancreatic cancer and for use in 3D visualizations. As of yet, no particular non-invasive tests for pancreatic cancer are accepted as definitive indicators of a lesion. False positives and false negatives are a constant concern for staging and pre-operative planning. CT provides solely anatomical details, but as seen here the use of contrast to delineate surrounding blood vessels was necessary. Using non-contrast CT for PET/CT fusion is a common practice for reasons that include concern for side effects of iodine contrast media, longer examination times, or higher radiation doses. Non-contrast CT has been brought into question for use in cancer staging because it does not delineate anatomical features as clearly as contrast enhanced CT [25], [26], [27], [28], [29], [30]. We used FDG PET as a metabolic indicator of malignant tissue, but contrast enhanced CT could be a second guide for denoting the tumor’s location using anatomical data.

The surface rendering method and program we used is only one option to display the data in 3D, but has certain advantages over maximum intensity projection (MIP) or volume rendering [31], [32], [33]. MIP and volume rendering display all of the intensity values of the data at once, as opposed to surface rendering, which only displays the surfaces of segmented regions. MIP displays the highest intensity value from the viewer’s point of view of volume data. This is a quick and easy method of viewing the volume, but lacks visual cues of depth. Volume rendering displays the intensities as well but provides a function for priority in displaying the data based on the viewer’s perspective, thus providing a better 3D feel. Without segmenting different regions in the data, all intensities are displayed at once, which can complicate the view. This is particularly true when organs have approximately the same intensities and are impacted upon each other. Surface rendering displays only the exterior of segmented regions without displaying the intensities of the data. While this only uses a fraction of the available information, it facilitates a much stronger visualization of the proximities of various organs. Mixing both types of visualizations would be useful for presenting different data sets in overlay, such as a surface rendering of CT data while using either MIP or volume rendering to overlay PET data. A drawback to this idea, in Amira 5, is that using overlapping transparent images is not possible without some way to establish a viewing priority.

Amira 5 is not the sole visualization package available for viewing PET/CT images both as either 2D slices or 3D rendering. Imaging instruments are bundle with proprietary imaging software that include software options for 3D post processing similar to Amira. Acquiring this software for use away from the instrument is generally as costly as Amira. Free software for is also available that can read image data and render it in 3D. 3D Slicer [34] is a well known free application for visualizing imaging data and has an active community for developing modules. Amira was chosen for this work for its modular design, which makes it easier to use, and for the it’s 3D modeling package to modify and export 3D objects.

One idea, suggested by a surgeon, would be to include visual warnings for adjacent structures that are close to the tumor. This could draw the surgeon’s attention to structures, such as blood vessels, near the tumor that could be of concern for unintended cutting. Another suggestion was to have the color of the vascular system be dependent on the theoretical oxygenation level in the blood.

The evaluating surgeons found the 3D visualizations to be useful tools for planning an operation, as a reference in the operating room during surgery, and as a reference for residents and assistants. Including PET images with the 3D rendering of the patient CT data had a positive influence on the surgeons’ perceived usefulness of the simulations. Using the contrast CT, instead of non-contrast CT, to generate a more complete blood vessel rendering improved the appeal of the 3D images for use by surgeons.

The next step in the development of this visualization system is to transfer the 3D models to the Simulation Open Framework Architecture (SOFA) [35]. By integrating a physical response model with the visualization, the surgeons will be able to interact directly with the models. A haptic interface will then be introduced to provide tactile feedback to the surgeons so that they can practice palpation of the tumor region *in silico* while planning the procedure. A touch-and-feel simulation of a specific patient could be a useful practice tool prior to making the first incision.
